# The Effect of Nitrogen Deposition on Plant Performance and Community Structure: Is It Life Stage Specific?

**DOI:** 10.1371/journal.pone.0156685

**Published:** 2016-06-02

**Authors:** Elise M. Tulloss, Mary L. Cadenasso

**Affiliations:** 1 University of California Davis, Department of Plant Sciences, Davis, California, United States of America; 2 La Salle High School, Yakima, Washington, United States of America; University of California Davis, UNITED STATES

## Abstract

Nitrogen (N) deposition is a key global change factor that is increasing and affecting the structure and function of many ecosystems. To determine the influence of N deposition on specific systems, however, it is crucial to understand the temporal and spatial patterns of deposition as well as the response to that deposition. Response of the receiving plant communities may depend on the life stage-specific performance of individual species. We focus on the California oak savanna because N deposition to this system is complex—characterized by hotspots on the landscape and seasonal pulses. In a greenhouse experiment, we investigated the relative influence of N deposition on plant performance during early growth, peak biomass, and senescent life stages across different soil types, light, and community compositions. To represent the community we used three grass species—a native, naturalized exotic, and invasive exotic. At early growth and peak biomass stages performance was measured as height, and shoot and root biomass, and at the senescent stage as seed production. Simulated N deposition 1) increased shoot biomass and height of the native and, even more so, the naturalized exotic during early growth, 2) positively affected root biomass in all species during peak biomass, and 3) had no influence on seed production at the senescent stage. Alone, N deposition was not a strong driver of plant performance; however, small differences in performance among species in response to N deposition could affect community composition in future years. In particular, if there is a pulse of N deposition during the early growth stage, the naturalized exotic may have a competitive advantage that could result in its spread. Including spatial and temporal heterogeneity in a complex, manipulative experiment provides a clearer picture of not only where N management efforts should be targeted on the landscape, but also when.

## Introduction

Nitrogen (N) deposition is an element of global change that affects plant community structure, typically driving increases in exotic species abundance and decreases in species richness [1–2]. While there has been a decline in N emissions in the US due to adoption of EPA Clean Air Act standards, N inputs to many ecosystems (including deposition) continue to increase depending on local and regional N sources [3–4]. Currently, many US ecosystems receive N deposition that exceeds critical levels for ecological impacts [5]. Here, we focus on the effects of N deposition in semi-arid savannas, using the California oak savanna as a model ecosystem. Deposition to this system and across the western US is expected to increase due mostly to agricultural and urban development [6]. Effects of N deposition on this system have included increases in exotic grass abundance, decreases in native species, decreases in sensitive lichen species, and changes in mycorrhizal communities [7]. To understand how N deposition may structure plant communities in this system, incorporating the characteristic spatial and temporal variability of this deposition into experimental tests is required.

Nitrogen deposition can be temporally and spatially complex [6]. Heterogeneity in the timing of N deposition may mediate its effects on plant communities. In systems that experience high variability in precipitation or distinct wet/dry cycles, N deposition is characterized by pulses that occur during discrete periods [8]. In seasonally dry systems, most deposition enters in dry form as gases and particles, but only becomes available to plants and soil microbes in pulses following precipitation events [9]. The pulsed nature of N deposition may have an important effect on plant community structure if the pulses correspond to crucial plant life-stages. In fact, plants in these strongly seasonal systems can possess adaptations enabling them to exploit pulsed N inputs [10]. Leger and Espelend [11] found that the strength and nature of interspecific competition varied across the plant life cycle suggesting that plants compete for particular external resources only during specific life stages. This means that a pulse of N deposition that occurs simultaneously with a period of strong interspecific competition for N should result in stronger effects of N deposition on the plant community than a pulse during a time of weak competition. In the semi-arid California savanna, we found that N deposition had little effect on germination and seedling establishment in three common grass species [12]. Because germination and seedling establishment are life stages in which interspecific competition is likely weak, this result was expected. Other life stages such as the rapid, early growth stage, the peak biomass/flowering stage, or the senescent, seed-setting stage may be timed to correspond to pulses of N deposition. Each of these three stages describes a different activity level and resource need for plants and therefore a potential for N deposition to impact plant functioning and competition. To evaluate whether a pulse in deposition at a particular life stage might be cause for concern for N deposition impacts to the plant community, differential impact of N inputs across life stages must be experimentally investigated. By holding N inputs constant, life stages where plant performance is highly sensitive to N can be identified.

In addition to temporal variation, plant communities can also be affected by variation in N deposition across space. Nitrogen deposition is spatially heterogeneous either because of the distribution of N emission sources or the distribution of landscape features that may capture that deposition differentially. Across California, N deposition rates can range from less than 2 kg/ha/yr to over 70 kg/ha/yr [6]. At these coarse scales, regional gradients in N deposition are created by emission sources, prevailing winds, and climate [13]. Deposition can also vary considerably at finer scales based on vegetation structure and land form [14]. The patchy vegetation of savanna landscapes leads to increased soil N beneath trees and shrubs, with deposition serving as a major contributor to these N hotspots [15]. In California savanna landscapes, solitary tree canopies embedded within a grassland matrix receive about double the amount of N deposition compared to the adjacent open grassland [16]. Because isolated tree canopies are structural discontinuities in the landscape, they may function similar to forest edges, and disrupt air flow, causing gases and particles to settle on leaf surfaces during dry periods and then rinse off during rainfall events [17]. This finer scale spatial heterogeneity in N deposition can be incorporated into experimental treatments to enhance realistic understanding of the impact of elevated N deposition on plant communities of systems that are structurally diverse.

At both coarse and fine scales, N deposition interacts with other factors creating complex suites of factors that may affect plants in combination. Empirical data on plant response to multiple interacting factors is often lacking in experiments [18]. In particular, there remains a need to incorporate the effects of heterogeneous soil and light environments into the study of N deposition effects on plant communities. Systems with less fertile soils may be more responsive to N deposition than those with more fertile soils. Low light levels, as a result of tree shade, may limit ground-layer plant responses to N deposition. Finally, the magnitude of N deposition may be important to its effect on the plant community. Nitrogen deposition often remains below a critical level, which may allow other ecological factors to override the effects of N [19]. Therefore, it is necessary to test a range of realistic field conditions and plant life stages in manipulative studies to understand N deposition effects on plant community structure.

In the greenhouse, we tested the effect of realistic levels of N deposition, relative to soil type and light availability, on native and exotic grass species mixtures. We used the California oak savanna as a model system to establish treatment conditions. Fertilization levels mimicked the amount of N deposition quantified under oak canopies and in the open grassland at sites that spanned a gradient in N deposition. To examine the impact of temporal heterogeneity in N deposition, we evaluated the response of plants across their adult growth life stages and identified specific stages particularly responsive to N additions. We hypothesized that: 1) the early growth stage is the most sensitive to N fertilization for all species because of high resource requirements at that time; 2) there is an overall positive effect of N on plants, but a greater response by exotic species when plants are grown in interspecific competition; 3) plants on lower fertility soils exhibit greater relative response to N additions; and 4) shaded conditions limit the response of these light-demanding grasses to N. The California oak savanna is an ideal system to test our hypotheses because 1) it experiences distinct wet and dry cycles that result in N inputs that are characteristically delivered to the ground layer in pulses, 2) there is evidence suggesting that N deposition to this system will increase invasive species abundance and threaten rare native species [20] but see [21, 3]) it is an important system in California covering approximately 1.5 million ha [22] and, therefore, is exposed to a gradient of regional deposition due to different emissions sources in the landscapes in which it is embedded, and 4) it is representative of the globally-distributed savanna biome that is the most spatially extensive biome in the world [23].

## Materials and Methods

### Study System

In the greenhouse, we established experimental conditions to mimic the heterogeneity in N deposition, soil type, and light availability characteristic of the savanna system. We had previously quantified the spatial and temporal heterogeneity in N deposition at several sites across the region, comparing deposition inputs beneath solitary canopy trees and the adjacent open grassland [16]. At the regional-scale we identified a gradient of N deposition with sites closest to human emission sources receiving greater N than remote sites. At a finer scale within savanna sites, oak understories were hotspots of N deposition, receiving about twice the inputs of the adjacent open grassland area. Overall, N deposition rates ranged from a low of 2 kg/ha/yr in the open at more remote sites to a high of 28 kg/ha/yr beneath canopies at more human-influenced sites [16]. These rates established our low and high fertilizer treatments. We also quantified the temporal heterogeneity of N deposition across the growing season. A pulse of N deposition was observed at all sites in the fall during the first significant precipitation event. A second pulse of equivalent size was observed in the early spring [16]. Because these pulses coincide with different plant life stages, N fertilizer addition was kept constant through the experiment to determine which life stage is most sensitive to N. Therefore, the documented spatial and temporal heterogeneity in N delivery to this system [16] was fundamental to the design of this experiment.

In addition to the heterogeneity of N deposition, soil types vary regionally and light availability varies locally. Soils were collected from three sites across the region for use in the experiment. These three sites represent a range of parent materials and soil fertility levels found across the California savanna system (S1 Fig, S1 Table). Light availability was experimentally manipulated to mimic the under-canopy/open-grassland mosaic characteristic of savannas.

The plant community of the California oak savanna is dominated by exotic annual grass species. Most exotic species in California are at the stage of naturalization, having been largely integrated into the ecosystem [24]. However, exotic species may have different characteristics depending on their current stage of invasion [25] and several new exotics are at a much earlier stage in the invasion process and are currently spreading across the region [26]. An annual invasive exotic and naturalized exotic, and a perennial native species were selected for the experiment. In California grasslands both the annual and perennial species experience a senescent phrase during the dry summer season [27].

### Experimental Design

In the experiment a full-factorial, split-plot design was used with light as a block factor. Factors were soil type, community mixture, light availability, and N fertilizer. The full model consisted of Plant performance metric ~ Nitrogen x Light x Soil type x Community mixture. This model was run for each life stage using different plant performance response variables for each life stage.

Soil was collected in June 2009 from Sierra Foothills Research and Extension Center (SFREC), Hopland Research and Extension Center (HREC), and San Joaquin Experimental Range (SJER). All three sites are located in the foothills of north-central California and they were chosen because of the range in soil nutrient concentrations and of parent materials (S1 Table). At each site a 1.5 by 1.5 meter area in open grassland was selected on a moderate slope (1–15%), with an overlying plant community representative of the oak savanna type and oak trees within 20 meters. In an attempt to preserve soil field conditions, soil was collected from two depths: 0–15 cm and 15–30 cm. The 0–30 cm depth was used because it captures about 80% of root biomass [28]. At the greenhouse, soil was mixed 2:1 with sterile sand to improve drainage and placed in 25 cm deep, 656 ml “Deepots” (Stuewe & Sons Inc., Tangent, OR) with paper towel at the bottom. Soil from the two depths was added to pots so that the 15–30 cm soil filled the bottom half of the pot and the 0–15 cm soil filled the top half. Prior to planting seeds, pots were leached with 50 ml of DI water daily for two weeks and germinating seeds from the soil seed bank were removed by hand.

Seeds of three common grasses–*Stipa pulchra*, a native perennial, *Hordeum murinum*, a naturalized exotic annual, and *Elymus caput-medusae*, an invasive exotic annual, were collected from Hopland Research and Extension Center (HREC) in Mendocino County in May and June 2009. Hereafter, the three species will be referred to as the native, naturalized exotic, and invasive exotic. All three species are C3, cool-season grasses that germinate and grow over the wet winter months, flower in the spring, and senesce by late spring or early summer. Seeds were collected from HREC because this location receives the lowest N deposition along the regional gradient– 2–5 kg/ha/yr. Thus, plants in the experiment were not pre-acclimated to high levels of N deposition. Seeds were planted in pots in single and multi-species community mixtures. All possible combinations of species were used for a total of seven community mixtures. Seeds were planted in a density resulting in 18 adult plants per pot. This density scales up to ~6500 individuals per square meter, a field density that is similar to communities containing native perennial grasses [29]. Equal numbers of individuals of each species per pot was attempted (18 monoculture, 9+9 biculture, 6+6+6 triculture).

To simulate the effect of the tree canopy on light availability, black shade cloth was installed over one half of the greenhouse. The greenhouse was oriented east-west and the shade cloth was installed on the east end. The shade cloth blocked 80% of sunlight, which approximated shade levels of the dominant tree species, *Quercus douglasii*, when the canopy is fully leafed out [30]. The color of the shade cloth is known to alter not only the amount of light but also the spectral quality of that light [31]. However, the influence of black shade cloth on spectral quality is unknown. Pots were centered beneath the shade cloth and non-shade cloth blocks to minimize daily fluctuations.

Fertilizer was applied weekly in 50 ml increments as an aqueous 1:1 ammonium nitrate solution. Nitrogen was added continuously during the fertilization treatment so that the difference in N supply within the small pots was retained throughout the experiment. This treatment was not intended to mimic the pulsed nature of N deposition. Two ammonium nitrate treatment levels were used to simulate the end members of the range of N deposition rates found across our study system: a low of 2 kg/ha/yr and a high of 28 kg/ha/yr (0.1 and 9 mg N/L respectively). Fertilizer was mixed with ¼ strength Hoagland’s solution to replace other nutrients potentially leached from soils [32]. Pots were also watered weekly or as needed, depending on greenhouse conditions, with 50 ml of DI water. During the experiment the greenhouse was maintained at a constant temperature (day/night temperatures of 24/12 degrees C) and a sulfur pot was used to treat powdery mildew. Fertilization/irrigation was initiated in October 2009. Irrigation was slowly decreased starting in late May 2010 to stimulate senescence.

Pots were completely randomized within one of two light blocks within the greenhouse. Ten replicate pots were assigned to each of 84 unique treatment combinations for a total of 840 pots harvested at each of 3 life stages: early growth, peak biomass, and senescence. Pots were spaced ~ 10 cm from one another to reduce inter-pot competition and were re-randomized within the light block following each harvest.

### Data Collection and Analysis

Pots were destructively harvested at 3 points over the course of the experiment: February, April, and June 2010, representing the early growth, peak biomass, and senescent life stages, respectively. For single-species communities, median height (cm), dry shoot biomass (grams per individual), and dry root biomass (grams per individual) were obtained. For mixed-species communities, the same information was collected for each species within the pot; however, root biomass could not be disentangled by species. Therefore, total biomass is not available for mixed species communities and root biomass data represents whole-community belowground allocation per pot. Root:shoot was calculated at the community level using the total shoot and total root biomass of the pot. If seeds were present, they were counted (seed number per individual) and weighed (seed milligrams per individual). For early growth and peak biomass stages, the response variables analyzed were height and shoot and root biomass. Seed production was the primary response variable for the senescent life stage because plant biomass had largely died back. Only the naturalized exotic produced seeds, therefore only community mixtures that contained this species were included in the analysis of senescent stage data.

For each of the three life stages, a 4-way MANOVA analysis was performed for height, shoot and root biomass from early and peak biomass life stage harvests and seed mass and number from the senescent life-stage harvest. MANOVA was used to avoid Type I error common with multiple comparisons of correlated variables [33] as well as to detect possible suites of traits critical in controlling N deposition response. MANOVAs were followed by separate univariate ANOVAs to identify which individual response variables were influenced by the experimental factors. Tukey post-hoc tests were used to identify significant differences among the multi-level factors (soil type, community mixture). Because the roots could not be separated by species, root:shoot data at the community level from early and peak biomass life stage harvests were analyzed using ANOVA. Unequal germination required that these response variables be scaled by the number of individuals of a species within a pot. All statistical tests were performed using JMP 5.1 (SAS Institute, Cary, North Carolina, USA).

The general statistical model was Plant performance metric ~ Nitrogen x Light x Soil type x Community mixture. For each life stage, the model was run for several specific plant traits used to measure plant performance. Specific plant trait data (height, biomass, etc.) were retained rather than aggregated into an index, such as an index of species response, so that any responses to N at different life stages could be linked to an actual trait. Despite this approach yielding complex results that may be difficult to interpret, using the performance data itself as the response variable allows us to address our central question of how response to N deposition changes over the course of the plant life cycle. Additionally, because our research question focuses on the influence of N fertilization on plant community structure, we use the Community mixture term in the model to assess competitive interactions among species in the experiment.

## Results

### Life Stage Response

**Fig 1:**
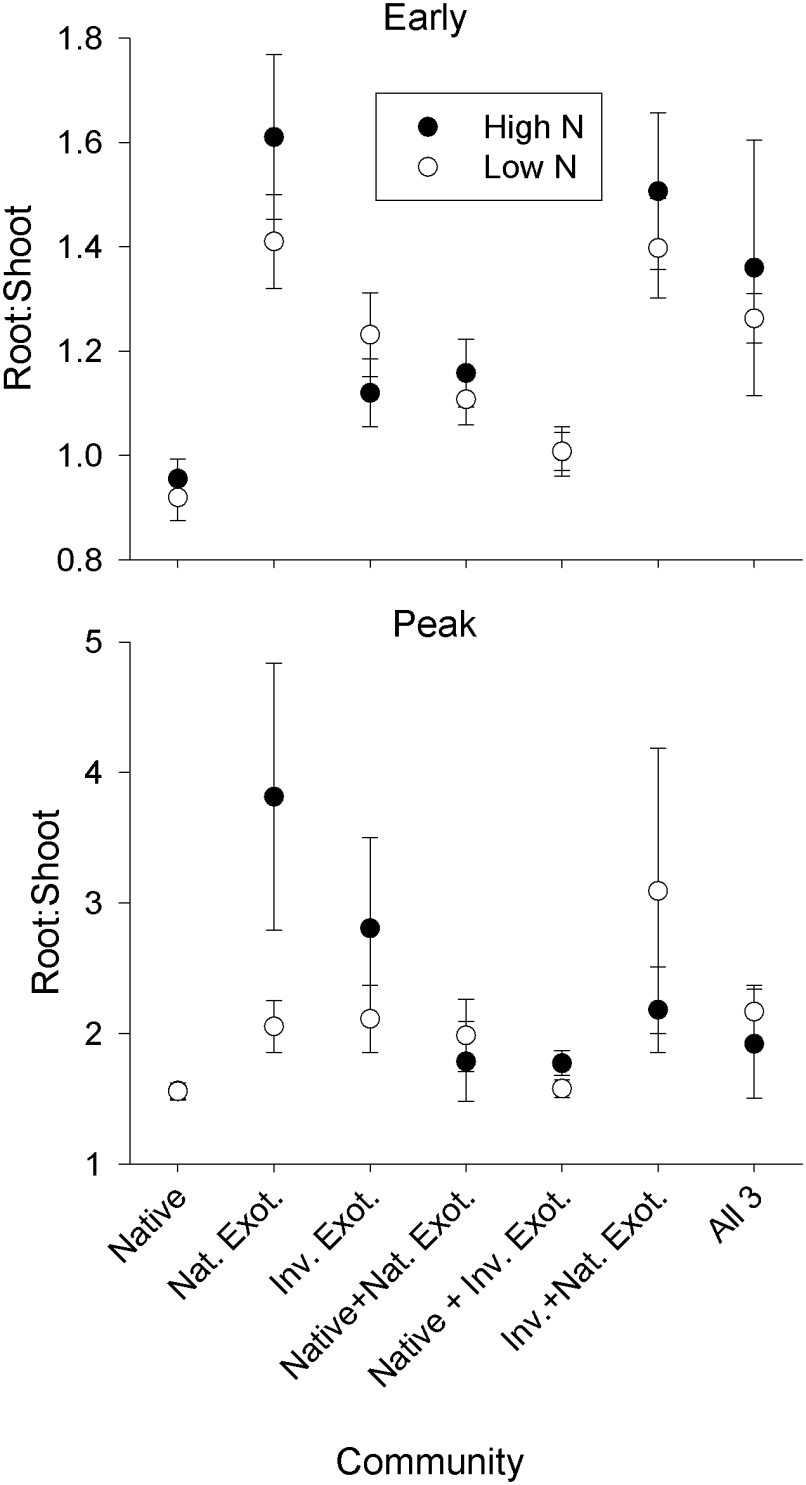
Root:shoot (± SE) at the community level for each community type under high versus low N fertilization during early growth and peak biomass stages. High N communities are represented by black symbols, low N by white symbols. Note difference in y-axis scales.

The effect of N fertilization on plant performance varied across life stages, shifting from a mostly aboveground response (height and shoot biomass) in early growth to a belowground response (root biomass) at peak biomass (Table 1). While this effect was general across all species, there were differences in how the species responded to N at each stage. There were no effects of N on root:shoot (Fig 1). For a complete summary of the statistical results, refer to Supporting Information (S2, S3, S4 and S5 Tables).

**Table 1:** Summary of univariate responses to N fertilization by each species during early growth and peak biomass life stages.

Life Stage	Species	Response Variable			
		Height	Shoot Biomass	Root Biomass	Root:Shoot
Early Growth	Native	+ *	ns	ns	
	Naturalized Exotic	+ *	+ **	+ *	
	Invasive Exotic	ns	ns	ns	
	Whole Community				ns
Peak Biomass	Native	ns	ns	+ **	
	Naturalized Exotic	ns	+ *	+ *	
	Invasive Exotic	ns	ns	+ *	
	Whole Community				ns

“+” indicates an increase in plant trait under high N,

“−” a decrease, and

“ns” indicates no response,

*P<0.05,

**P<0.01

**Fig 2:**
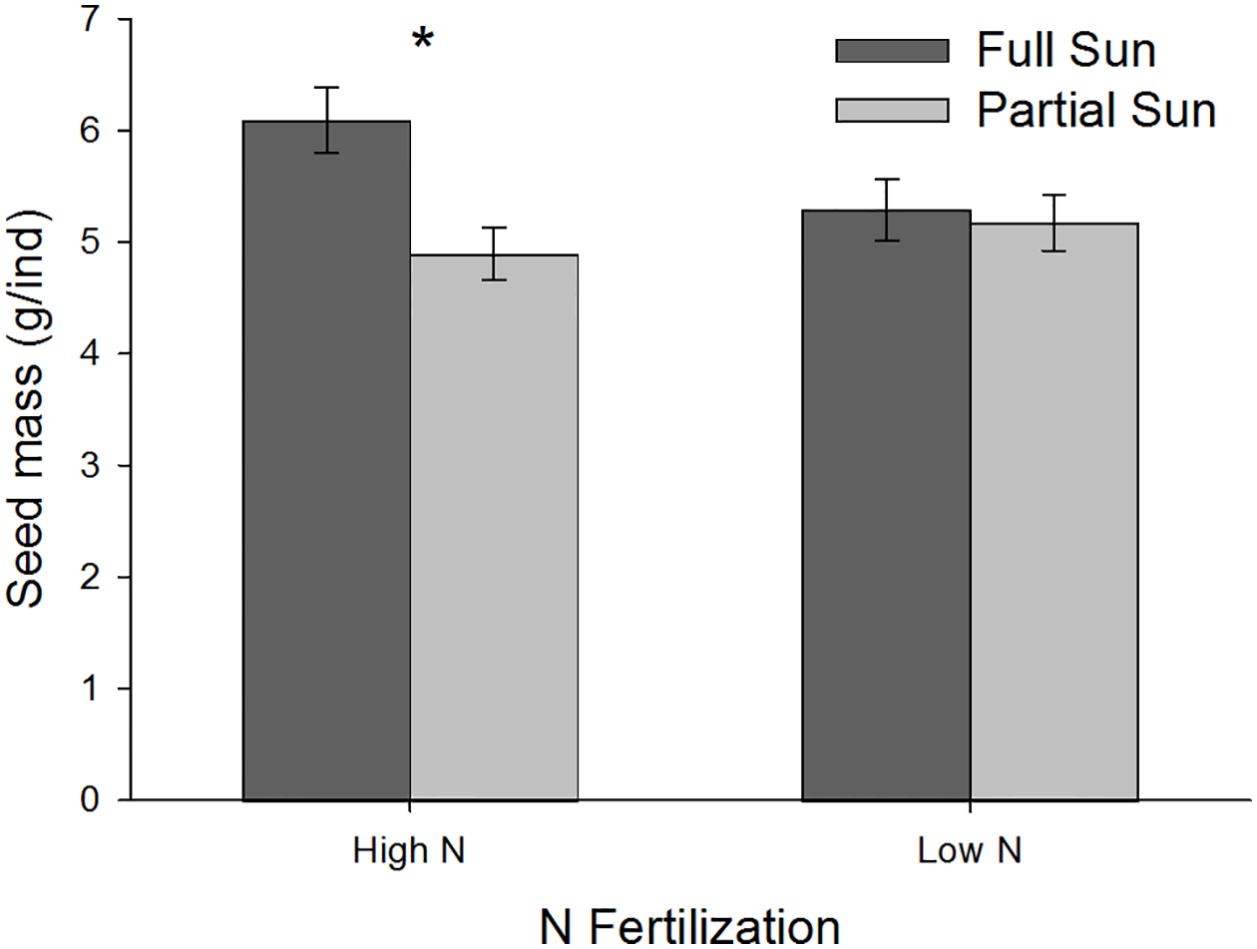
Mean mass (±SE) of naturalized exotic seeds produced by senescent plants grown under high versus low N fertilizer in contrasting light environments. * Indicates significant difference at P<0.05.

During early growth, the naturalized exotic responded to N fertilization through increased height, shoot and root biomass (N, Wilks’ Lambda F_4,423_ = 5.170, P = 0.001). The invasive exotic showed no significant main effect of N (N, P>0.05), and the native had no significant response to N at the multivariate level, but univariate analysis showed increased height under high N (N, F_4,424_ = 4.127, P = 0.0428). In contrast to early growth, at peak biomass the invasive exotic and native responded to high N through greater root biomass (invasive: N, F_1,430_, P = 0.0335; native: N, Wilks’ Lambda F_4,423_ = 4.465, P = 0.0042), and the naturalized exotic through increased shoot and root biomass (N, Wilks’ Lambda F_4,423_ = 2.928, P = 0.0335). At the senescent life stage, live biomass was less than the earlier two harvests for all species, indicating that the irrigation reduction successfully stimulated senescence (S2 Table). The performance variables examined during the senescent life stage—seed number and mass of the naturalized exotic—did not respond to N fertilization alone (N, P>0.05). However, seed response to light was mediated by N (N x Light, F_2,236_ = 4.0526, P = 0.0186). Under high N, plants growing in full sun exhibited increased seed mass compared to partial sun, but under low N, there was no difference between light treatments (Fig 2).

### Community Mixture Effects

**Fig 3:**
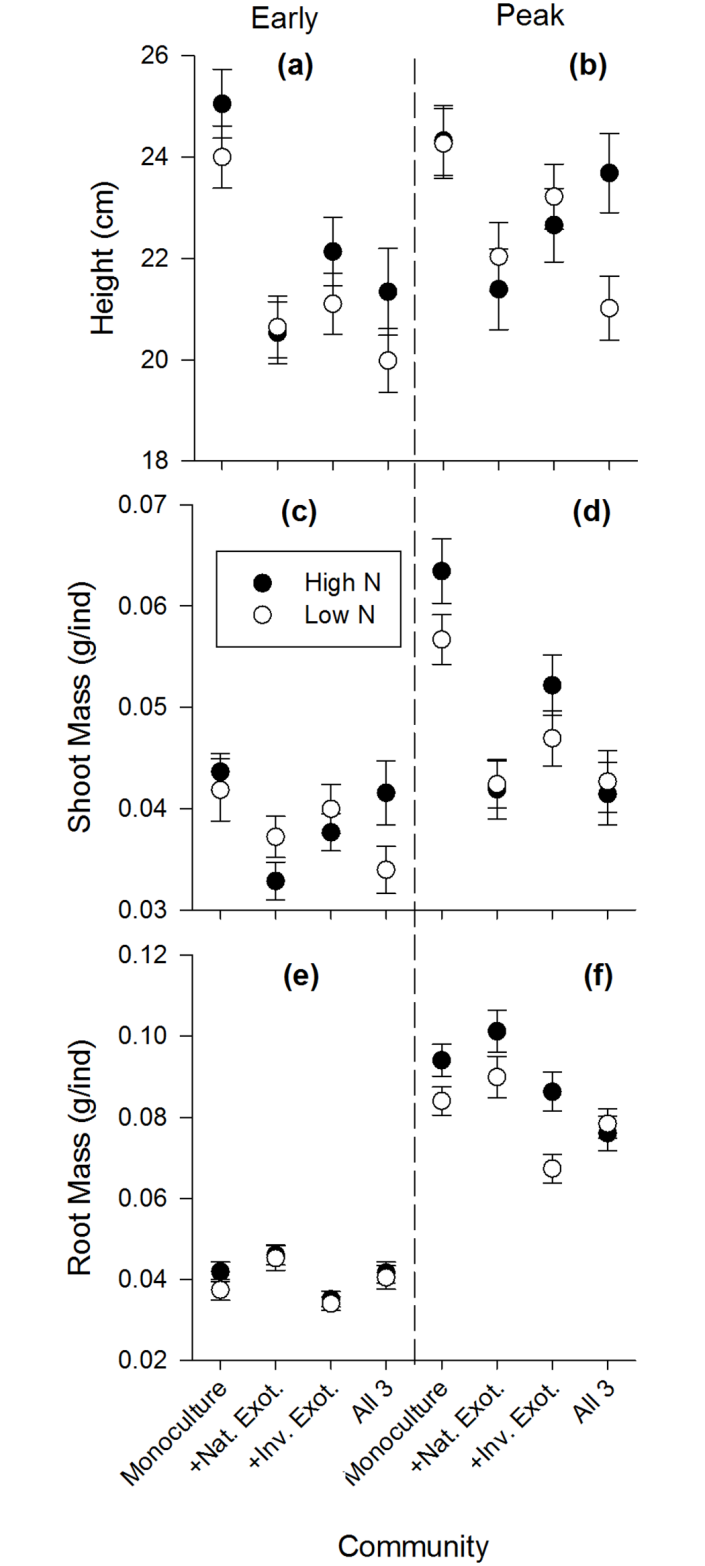
Natives species performance during early growth and peak biomass life stages in each community mixture. High N communities are black symbols, low N are white symbols. Plant performance is measured as the mean (±SE) of: shoot mass grams per individual (g/ind), root mass grams per individual (g/ind), and height (cm).

**Fig 4:**
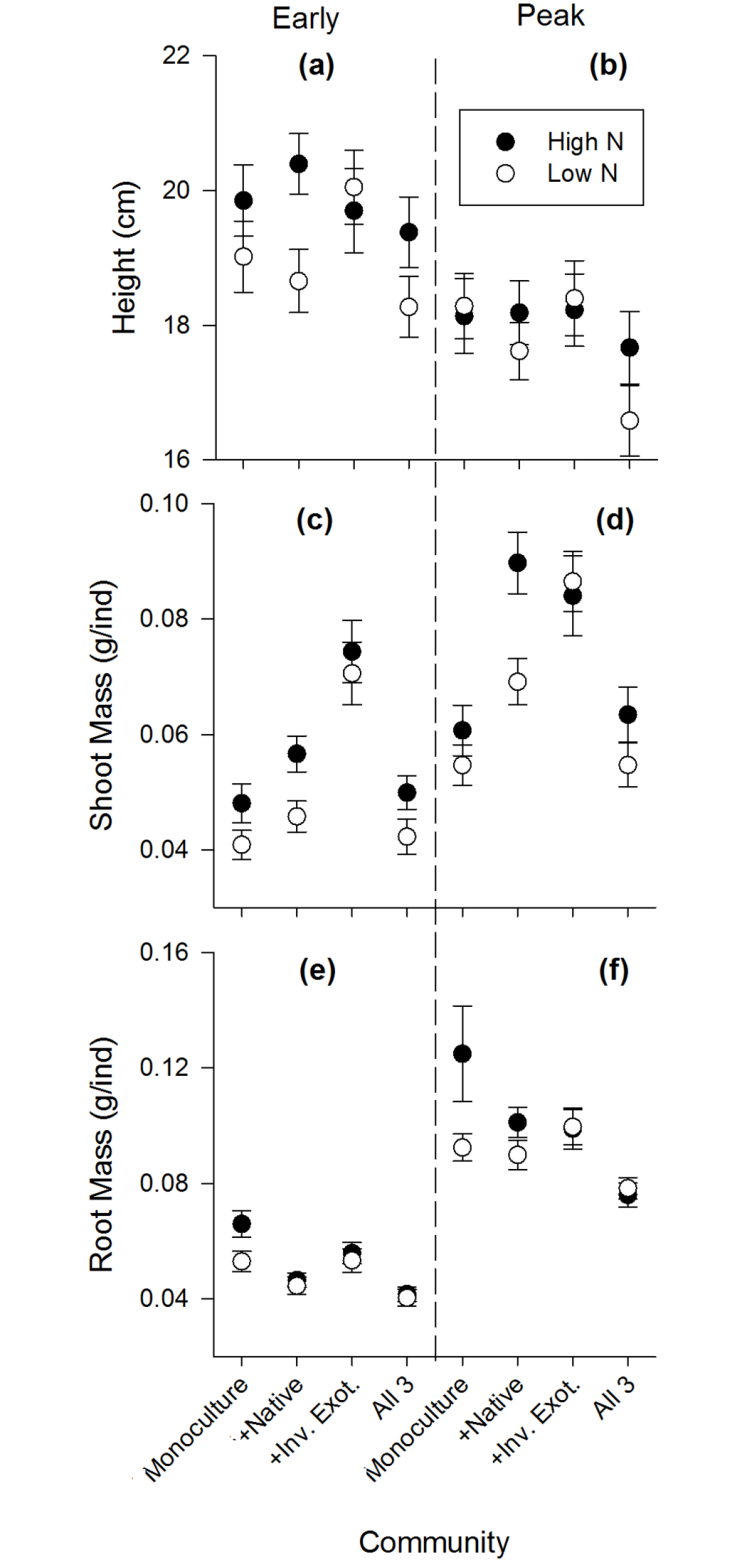
Naturalized exotic species performance during early growth and peak biomass life stages in each community mixture. High N communities are black symbols, low N are white symbols. Plant performance is measured as the mean (±SE) of: shoot mass grams per individual (g/ind), root mass grams per individual (g/ind), and height (cm).

**Fig 5:**
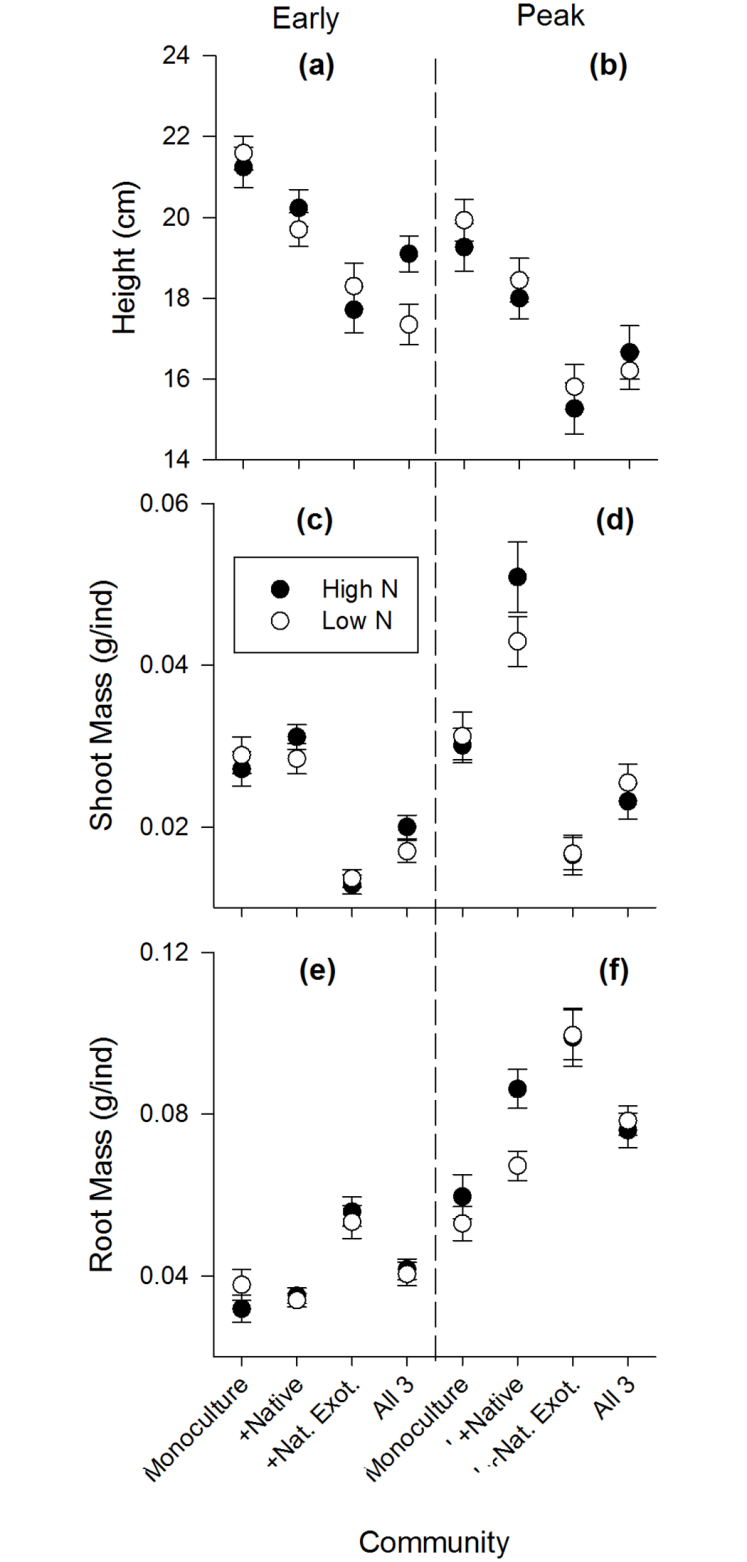
Invasive exotic species performance during early growth and peak biomass life stages in each community mixture. High N communities are black symbols, low N are white symbols. Plant performance is measured as the mean (±SE) of: shoot mass grams per individual (g/ind), root mass grams per individual (g/ind), and height (cm).

Inter- and intraspecific competition were important factors determining plant performance in the experiment. During early growth and peak biomass, all species showed significantly different performance depending on the community mixture they grew in (Figs 3–5). For aboveground traits, the native performed best in communities with only intraspecific competition (Fig 3A–3D), the naturalized exotic performed best when grown with one competitor species (Fig 4A–4D), and the invasive performed best alone or in mixture with the native species only (Fig 5A–5D). In contrast, root mass of naturalized exotic communities was greatest where the naturalized exotics were growing alone, especially under high N (Fig 4E and 4F). Communities containing native and/or invasive exotics had the greatest root mass where natives and invasive exotics were growing with naturalized exotics (Figs 3E and 3F and 5E and 5F). This suggests naturalized exotics contributed most of the root biomass in mixed species communities.

Nitrogen fertilization only affected competitive interactions (a significant N x Community interaction) in a few specific cases during early growth and peak biomass. There was no effect of N on competitive interactions during the senescent stage. During early growth, shoot mass of the native growing with the naturalized exotic was significantly lower under high N, but shoot mass of the native growing with all 3 species was significantly greater under high N (Fig 3C). During peak biomass, the native growing in 3-species mixture had significantly greater height under high N (Fig 3B). Also during peak biomass, the naturalized exotic growing alone had significantly greater root mass under high N (Fig 4F), and communities with the native and invasive exotic growing together had greater root mass under high N although this was only marginally significant for the native (Figs 3F and 5F).

### Abiotic Effects

The response to soil type and light availability was typically not mediated by the N fertilization treatment, except in a few specific cases. All species responded as hypothesized to the soil fertility gradient, with increasing biomass and height from low to high fertility soil (S2 and S3 Tables; S3 Fig). Seed production of the naturalized exotic also increased with soil fertility. A significant Soil x N interaction was observed for native species during early growth (N x Soil, Wilks’ Lambda F_8, 842_ = 2.184, P = 0.0283). Univariate Tukey post-hoc tests showed native species had greater height on high fertility soils receiving high N compared to low N. No differences between high and low fertilizer were observed for the other two soils.

Under partial sun, all plants showed increased height, but other responses to the light treatment were life stage and species-specific. During peak biomass, all species had lower root biomass in partial sun, while the native also had greater shoot biomass (S3 Table). During early growth, naturalized and invasive exotic plants growing in partial sun had greater height under high N (Naturalized exotic: N x Light, F_1, 420_ = 4.338, P = 0.0244; Invasive exotic: N x Light, F_1,427_ = 4.542, P = 0.0336).

## Discussion

Nitrogen deposition into N limited systems can have dramatic effects on plant community structure. Deposition to the California oak savanna is delivered heterogeneously in both time and space due to rainfall patterns and distributions of tree canopies. How this deposition influences plant community structure may depend on both the plant life stage of the receiving community and other environmental factors.

### Sensitivity of Early Growth Stage

Nitrogen deposition is typically delivered to the California oak savanna in pulses [34]. The first rains of the wet season in the fall rinse accumulated dry deposition from surfaces and mobilize soil microbes. A similar pulse can occur later in the wet season if there is a prolonged dry period, which is often the case in drought-prone savanna. Thus, plants in the early growth stage are likely to receive pulses of N deposition coinciding with a time of rapid vegetative growth requiring high external resources [35]. We found that the early growth stage was the most sensitive life stage to N fertilization; at later stages there were fewer effects of N on plants and N had no effect on seed production. These results suggest that a pulse of N deposition during early growth may have a greater effect on plants than N deposition at later life stages. This finding is consistent with research in a desert annual community demonstrating that the rapid vegetative growth stage is when plants are most sensitive to the impacts of resource level on competition [36].

Variation in phenology among species may influence the ability of species to take advantage of a pulse of N deposition. Though this difference in phenology may only be on the order of a few days, rapid early growth could lead to a significant competitive advantage by species that become active first. In California oak savannas, naturalized exotic annual grasses tend to enter the early growth stage earlier than other grass species and senesce earlier the spring [37]. The native, a long-lived perennial, typically emerges from dormancy slightly later than the naturalized exotic and grows much more slowly throughout the season [38]. The delay in other plant species may make them less likely to be able to respond to N deposition pulses early in the season and put them at a disadvantage compared to naturalized exotics. We assumed constant phenology across our three species but in reality the invasive exotic tended to enter the early growth stage later than the other two species and was active later into the spring [37]. The delay of early growth onset in the invasive exotic may result in this species being less able to respond to N deposition pulses early in the season compared to the naturalized exotic and native species.

### Competitive Hierarchies and Biomass Allocation Pattern Maintained

Studies investigating competition between native perennial and exotic annual grasses in California’s Central Valley have consistently concluded that exotic annual grasses reduce the performance of the native grasses (e.g., [39–40]). Nitrogen fertilization of these systems increases productivity [41] and elevated N is expected to favor exotic species over native species shifting species composition towards exotic annual grasses [1, 42–45]. However, under the realistic N deposition scenario imposed here, we found few instances in which N fertilization affected the outcome of inter- and intra- specific competition (N x Community interaction). There was only one finding—decreased shoot biomass of the native in competition with the naturalized exotic—where N fertilization clearly favored an exotic over the native. This finding suggests that high N deposition may lead to a general increase in productivity as previously determined [41], but, in contrast to other studies, high N deposition may lead to no change in the relative abundance of common grass species. It is possible that N is not the limiting resource affecting interactions among these species. For example, the invasive species, *Elymus caput-medusae*, has been shown to be a successful invader in both low and high resource ecosystems [46]; indicating N deposition may not exacerbate its spread. In addition, all of the species used in our experiment were grasses and N deposition may have more of an impact on the abundance of grasses relative to forbs. Under realistic levels of N, abundance of grasses increased relative to forbs in a field experiment [46].

We expected communities in our high N treatment to decrease belowground allocation. This general expectation was confirmed in a controlled experiment with a California invasive grass that found shoot biomass responded to elevated N while root biomass and physiological traits did not [47]. In our experiment, however, we did not find evidence that N deposition is causing shifts in biomass allocation patterns. Both shoot and root biomass increased in the high N treatment resulting in no significant effect of N on root:shoot. Because limitation by multiple resources including N, phosphorous, potassium, sulfur, and water has commonly been found in these systems [41], we hypothesize that the high N treatment may have facilitated greater root exploration of soil, to capture a different limiting belowground resource.

### Nitrogen Response and Abiotic Factors

The abiotic factors we examined—soil type and light availability—did not interact with N as we hypothesized. We expected plant response to N fertilization to be less on high fertility soil compared to low fertility soil and we expected plant response to N fertilization to be less under low light conditions compared to high light conditions. Responses to N fertilization, however, were not consistent with these expectations; significant N x Light or Soil interactions were specific to the species or life stage. In particular, performance of the native grass to the high N fertilization treatment was greater on high fertility soil compared to low fertility soil. This suggests that limitations other than N drove performance on the lower fertility soils. These complex interactions emphasize the importance of environmental heterogeneity in controlling plant community response to N deposition [48] and provides an important caution to predicting the effects of N deposition based on soil type—N deposition to low fertility soils may not always result in increased productivity because these soils may also be of poor quality in other growth-limiting factors (e.g. drainage, salinity, other nutrients) [41].

The chemical form of the N used for the fertilization treatment may have affected plant response. A 1:1 ratio of ammonium- to nitrate-N was used, but this ratio is not typical in deposition to California oak savannas. This ratio is highly variable from site to site within the region with some sites being dominated by ammonium-N and others dominated by nitrate-N [16]. A 1:1 ratio represents an average, but we recognize the potential limitations of extrapolating our results to sites with different ratios.

Light limitation imposed by the tree canopy may be an important controller of plant performance in California oak savannas [49–50]. In the partial sun treatment imposed to mimic environments under the canopy of an oak, we expected plant performance to be lower. Surprisingly, we found that partial sun did not limit performance and, in fact, height and biomass were greater under this treatment. Nitrogen fertilization effects were sometimes expressed only in the partial sun treatment. This result is important because the understory is a deposition hotspot [16] and low light availability under the canopy may not offset the impact of enhanced N deposition on the plant community. Thus, in the field, savanna understory plants may be just as affected, if not more so, by N deposition.

## Conclusions

To determine the influence of N deposition on specific systems, it is crucial to understand the temporal and spatial patterns of deposition as well as the response to that deposition. Response of the receiving plant communities may depend on the life stage-specific performance of individual species. Nitrogen deposition must be considered in the context of multiple interacting factors. Alone, N may not always be a strong driver of plant community structure and dynamics, but taking into account the time of year, the competitive environment, and two key abiotic factors—soil type and light—we identified particular scenarios where N does affect the plant community. For example, places with high N deposition (>28 kg/ha/yr) and shaded microenvironments may be more susceptible to N deposition effects, and those effects may be concentrated during the early growth life stage of the plant. Obviously this is a controlled experiment with a small subset of species from the oak savanna community and responses found in this situation may not match responses in the natural environment. Identifying the specific functional and/or phenological traits affected by N could be a key piece of information to help understand which species and plant communities may be most sensitive to chronic enhanced N deposition. Including these multiple factors into a complex, manipulative experiment provides a clearer picture of not only where N management efforts should be targeted on the landscape, but also when.

## Supporting Information

S1 Fig Locations in California of three sites used to collect soil and seed (HREC only) for the greenhouse experiment. Clockwise from top-left: Hopland Research and Extension Center (HREC) in Mendocino County, Sierra Foothills Research and Extension Center (SFREC) in Yuba County, and San Joaquin Experimental Range (SJER) in Madera County.
(JPG)

S2 Fig Mean aboveground biomass (±SE) at each life stage averaged across all treatments. (TIF)

S3 Fig Mean plant height (±SE) of natives during a) early growth stage and b) peak biomass stage under contrasting soil types and N fertilizer levels. * Indicates significant difference between high and low N for that treatment combination (P<0.05).
(TIF)

S4 Fig Early growth response of mean height (±SE) to Light x N interaction in a) Naturalized exotic and b) Invasive exotic. Asterisks (*) indicate significant differences between full and partial sun treatments for that treatment combination (P<0.05).
(TIF)

S1 File Raw data used for all analyses and figures presented in manuscript. (XLSX)

S1 Table Comparison of soil from three sites used in experiment. (DOCX)

S2 Table 4-way ANOVA and MANOVA (Nitrogen x Soil Type x Light x Community) results for performance of individual plant species during early growth. Significant factors (P<0.05) are in bold. All possible interactions were included in statistical model, but non-significant 3 and 4-way interactions are not shown.
(DOCX)

S3 Table 4-way ANOVA and MANOVA (Nitrogen x Soil x Light x Community) results for performance of individual plant species during peak biomass. Significant factors (P<0.05) are in bold. All possible interactions were included in statistical model, but non-significant 3 and 4-way interactions are not shown.
(DOCX)

S4 Table 4-way ANOVA (Nitrogen x Soil Type x Light x Community) results for whole-community root:shoot during the early growth and peak biomass life stages. Significant factors (P<0.05) are in bold. All possible interactions were included in statistical model, but non-significant 3 and 4-way interactions are not shown.
(DOCX)

S5 Table 4-way ANOVA and MANOVA (N x Soil Type x Light x Community) results for naturalized exotic seed mass and number during the senescent stage. Significant factors (P<0.05) are in bold. All possible interactions were included in statistical model, but non-significant 3 and 4-way interactions are not shown.
(DOCX)
