# Supplementary figures and images for: The Effect of Nitrogen Deposition on Plant Performance and Community Structure: Is It Life Stage Specific?
Source: PLoS One. 2016 Jun 2;11(6):e0156685. doi: 10.1371/journal.pone.0156685 (PMC4890792; doi:10.1371/journal.pone.0156685)

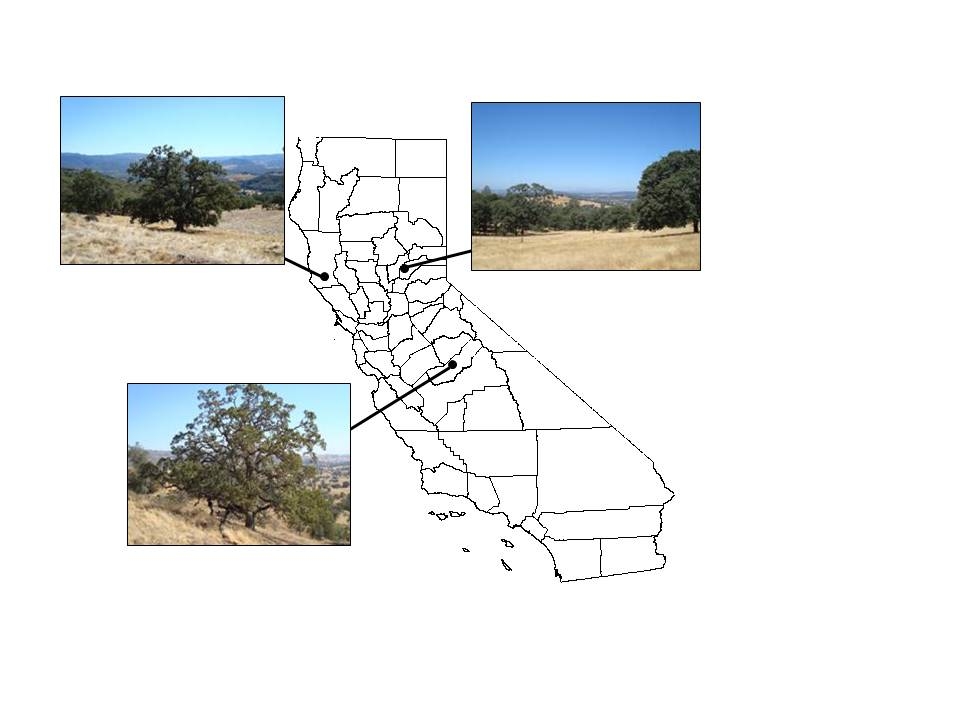

Supplement: S1 Fig — Clockwise from top-left: Hopland Research and Extension Center (HREC) in Mendocino County, Sierra Foothills Research and Extension Center (SFREC) in Yuba County, and San Joaquin Experimental Range (SJER) in Madera County. (JPG) [file pone.0156685.s001.jpg]
