# Supplementary material for: The Effect of Nitrogen Deposition on Plant Performance and Community Structure: Is It Life Stage Specific?
Source: PLoS One. 2016 Jun 2;11(6):e0156685. doi: 10.1371/journal.pone.0156685 (PMC4890792; doi:10.1371/journal.pone.0156685)
Supplement: S1 Table — (DOCX) [file pone.0156685.s006.docx]

**S1 Table. Comparison of soil from three sites used in experiment**

| Soil Factor | Site | | |
| --- | --- | --- | --- |
|  | SFREC | HREC | SJER |
|  |  |  |  |
| Total N (g kg^-1^)^a^ | 3 to 5 | 1.5 to 3 | 1 to 1.5 |
| Texture^a^ | Silty clay loam | Loam | Sandy loam |
| Water-holding Capacity (%)^b^ | 66 | 64 | 54 |
| Parent Material^a^ | Metamorphosed basalt | Sandstone/greywacke | Dissolved granite |
| Precipitation (cm)^c^ | 74 | 94 | 56 |
| N deposition (kg ha^-1^ yr^-1^)^d^ | 6 to 10 | 2 to 5 | 7 to 13 |

^a^Dahlgren et al. 2003

^b^Tulloss *unpublished data*

^c^California Irrigation Management Systems 2009

^d^Throughfall/bulk N deposition from canopy and open areas (Tulloss & Cadenasso 2015)
