# Supplementary material for: The Effect of Nitrogen Deposition on Plant Performance and Community Structure: Is It Life Stage Specific?
Source: PLoS One. 2016 Jun 2;11(6):e0156685. doi: 10.1371/journal.pone.0156685 (PMC4890792; doi:10.1371/journal.pone.0156685)
Supplement: S2 Table — Significant factors (P<0.05) are in bold. All possible interactions were included in statistical model, but non-significant 3 and 4-way interactions are not shown. (DOCX) [file pone.0156685.s007.docx]

**S2 Table. 4-way ANOVA and MANOVA (Nitrogen x Soil x Light x Community) F statistics for performance of individual plant species during early growth.**

|  |  | ANOVA | | | | MANOVA | |
| --- | --- | --- | --- | --- | --- | --- | --- |
| Species | Factor | df | Height (cm) | Shoot Mass (g/ind.) | Root Mass  (g/ind.) | df | Wilks Lambda |
| Native |  |  |  |  |  |  |  |
|  | Nitrogen | 1 | 4.127* | 0.239 | 1.779 | 4 | 2.216 |
|  | Light | 1 | 0.156 | 0.152 | 1.409 | 4 | 0.493 |
|  | Soil | 2 | 66.491*** | 94.902*** | 98.439*** | 8 | 50.187*** |
|  | Community | 3 | 20.143*** | 5.314** | 10.259*** | 12 | 11.781*** |
|  | Nitrogen x Light | 1 | 0.473 | 1.761 | 0.040 | 4 | 1.429 |
|  | Nitrogen x Soil | 2 | 5.832** | 1.965 | 0.193 | 8 | 2.184* |
|  | Nitrogen x Community | 6 | 0.657 | 3.774* | 0.346 | 12 | 1.677 |
|  | Light x Soil | 2 | 0.076 | 0.292 | 0.032 | 8 | 0.181 |
|  | Light x Community | 3 | 2.7291* | 0.275 | 2.410 | 12 | 1.779 |
|  | Soil x Community | 6 | 1.141 | 4.356*** | 4.900*** | 24 | 2.953*** |
| Naturalized Exotic |  |  |  |  |  |  |  |
|  | Nitrogen | 1 | 5.814* | 13.238*** | 5.181* | 4 | 5.170** |
|  | Light | 1 | 11.214*** | 0.314 | 3.976* | 4 | 9.169*** |
|  | Soil | 2 | 28.753*** | 105.383*** | 156.500*** | 8 | 59.341*** |
|  | Community | 3 | 1.725 | 42.182*** | 21.451*** | 12 | 25.326*** |
|  | Nitrogen x Light | 1 | 4.394* | 6.938** | 0.366 | 4 | 2.683* |
|  | Nitrogen x Soil | 2 | 0.793 | 0.420 | 0.701 | 8 | 0.739 |
|  | Nitrogen x Community | 6 | 1.601 | 0.437 | 1.630 | 12 | 1.285 |
|  | Light x Soil | 2 | 0.995 | 1.408 | 0.200 | 8 | 1.093 |
|  | Light x Community | 3 | 1.514 | 0.832 | 1.629 | 12 | 1.693 |
|  | Soil x Community | 6 | 5.923*** | 24.190*** | 5.460*** | 24 | 8.718*** |
|  | Nitrogen x Light x Soil | 2 | 1.673 | 3.645* | 1.416 | 8 | 1.442 |
|  | Nitrogen x Soil x Community | 6 | 0.778 | 2.277* | 1.560 | 24 | 1.168 |
| Invasive Exotic |  |  |  |  |  |  |  |
|  | Nitrogen | 1 | 1.641 | 0.855 | 0.964 | 4 | 0.9072 |
|  | Light | 1 | 9.944** | 0.108 | 0.861 | 4 | 3.7954* |
|  | Soil | 2 | 57.400*** | 40.845*** | 7.891*** | 8 | 26.2738*** |
|  | Community | 3 | 29.879*** | 58.237*** | 1.154 | 12 | 22.7537*** |
|  | Nitrogen x Light | 1 | 4.542* | 1.379 | 0.834 | 4 | 3.1111* |
|  | Nitrogen x Soil | 2 | 0.580 | 1.024 | 0.658 | 8 | 0.8976 |
|  | Nitrogen x Community | 6 | 3.068* | 1.216 | 0.625 | 12 | 1.4035 |
|  | Light x Soil | 2 | 0.419 | 0.663 | 1.254 | 8 | 0.7992 |
|  | Light x Community | 3 | 0.898 | 0.826 | 1.073 | 12 | 1.0304 |
|  | Soil x Community | 6 | 7.908*** | 8.565*** | 1.240 | 24 | 5.1328*** |

MANOVA used Wilk’s Lambda to calculate F statistic and p-values. All possible interactions were included in statistical model, but non-significant 3 and 4-way interactions are not shown.

*P<0.05

**P<0.01

***P<0.001
