# Supplementary material for: The Effect of Nitrogen Deposition on Plant Performance and Community Structure: Is It Life Stage Specific?
Source: PLoS One. 2016 Jun 2;11(6):e0156685. doi: 10.1371/journal.pone.0156685 (PMC4890792; doi:10.1371/journal.pone.0156685)
Supplement: S3 Table — Significant factors (P<0.05) are in bold. All possible interactions were included in statistical model, but non-significant 3 and 4-way interactions are not shown. (DOCX) [file pone.0156685.s008.docx]

**S3 Table. 4-way ANOVA and MANOVA (Nitrogen x Soil x Light x Community) F statistics for performance of individual plant species during peak biomass.**

|  |  | ANOVA | | | | MANOVA | |
| --- | --- | --- | --- | --- | --- | --- | --- |
| Species | Factor | df | Height (cm) | Shoot Mass (g/ind.) | Root Mass  (g/ind.) | df | Wilks Lambda |
| Native |  |  |  |  |  |  |  |
|  | Nitrogen | 1 | 1.118 | 2.052 | 13.021*** | 4 | 4.465** |
|  | Light | 1 | 269.889*** | 12.414*** | 14.575*** | 4 | 94.916*** |
|  | Soil | 2 | 55.595*** | 45.178*** | 70.140*** | 8 | 41.199*** |
|  | Community | 3 | 9.807*** | 22.459*** | 12.431*** | 12 | 12.342*** |
|  | Nitrogen x Light | 1 | 2.879 | 0.001 | 0.219 | 4 | 1.151 |
|  | Nitrogen x Soil | 2 | 4.287* | 0.825 | 0.160 | 8 | 2.002 |
|  | Nitrogen x Community | 6 | 4.620** | 1.141 | 2.715* | 12 | 3.173*** |
|  | Light x Soil | 2 | 2.266 | 0.156 | 0.221 | 8 | 0.881 |
|  | Light x Community | 3 | 2.170 | 0.699 | 0.361 | 12 | 1.176 |
|  | Soil x Community | 6 | 7.231*** | 4.704*** | 4.815*** | 24 | 4.626*** |
|  | Light x Soil x Community | 6 | 2.981** | 0.829 | 0.156 | 24 | 1.366 |
| Naturalized Exotic |  |  |  |  |  |  |  |
|  | Nitrogen | 1 | 0.999 | 6.348* | 5.043* | 4 | 2.928* |
|  | Light | 1 | 286.149*** | 1.724 | 5.526* | 4 | 107.667*** |
|  | Soil | 2 | 27.117*** | 29.850*** | 40.885*** | 8 | 23.970*** |
|  | Community | 3 | 3.395* | 19.168*** | 6.887*** | 12 | 10.455*** |
|  | Nitrogen x Light | 1 | 4.338* | 2.697 | 0.230 | 4 | 1.917 |
|  | Nitrogen x Soil | 2 | 1.093 | 0.555 | 1.841 | 8 | 1.043 |
|  | Nitrogen x Community | 6 | 1.489 | 2.575* | 2.076 | 12 | 1.868* |
|  | Light x Soil | 2 | 0.430 | 0.898 | 0.333 | 8 | 0.765 |
|  | Light x Community | 3 | 0.164 | 0.074 | 1.691 | 12 | 0.656 |
|  | Soil x Community | 6 | 1.186 | 7.088*** | 1.486 | 24 | 3.150*** |
|  | Light x Soil x Community | 6 | 2.163* | 1.120 | 0.416 | 24 | 1.098 |
| Invasive Exotic |  |  |  |  |  |  |  |
|  | Nitrogen | 1 | 0.652 | 0.282 | 4.551* | 4 | 1.033 |
|  | Light | 1 | 215.730*** | 3.981* | 4.242* | 4 | 97.706*** |
|  | Soil | 2 | 22.998*** | 15.642*** | 44.357*** | 8 | 21.165*** |
|  | Community | 3 | 32.300*** | 49.911*** | 29.878*** | 12 | 37.231*** |
|  | Nitrogen x Light | 1 | 0.092 | 0.006 | 1.544 | 4 | 0.300 |
|  | Nitrogen x Soil | 2 | 2.096 | 1.544 | 0.358 | 8 | 0.901 |
|  | Nitrogen x Community | 6 | 0.756 | 1.474 | 1.632 | 12 | 1.802 |
|  | Light x Soil | 2 | 4.387* | 2.212 | 0.024 | 8 | 1.943 |
|  | Light x Community | 3 | 3.881** | 2.283 | 0.779 | 12 | 2.696** |
|  | Soil x Community | 6 | 3.956*** | 1.122 | 3.748** | 24 | 2.808*** |
|  | Light x Soil x Community | 6 | 1.795 | 3.433** | 0.243 | 24 | 1.647* |

MANOVA used Wilk’s Lambda to calculate F statistic and p-values. All possible interactions were included in statistical model, but non-significant 3 and 4-way interactions are not shown.

*P<0.05

**P<0.01

***P<0.001
