# Supplementary material for: The Effect of Nitrogen Deposition on Plant Performance and Community Structure: Is It Life Stage Specific?
Source: PLoS One. 2016 Jun 2;11(6):e0156685. doi: 10.1371/journal.pone.0156685 (PMC4890792; doi:10.1371/journal.pone.0156685)
Supplement: S4 Table — Significant factors (P<0.05) are in bold. All possible interactions were included in statistical model, but non-significant 3 and 4-way interactions are not shown. (DOCX) [file pone.0156685.s009.docx]

**S4 Table. 4-way ANOVA (Nitrogen x Soil x Light x Community) results for whole-community root:shoot during the early growth and peak biomass life stages.**

| Life Stage | Factor | df | F | P |
| --- | --- | --- | --- | --- |
| Early Growth |  |  |  |  |
|  | Nitrogen | 1, 749 | 1.001 | 0.3174 |
|  | Light | 1, 749 | 0.008 | 0.9266 |
|  | Soil | 2, 749 | 7.163 | **0.0008** |
|  | Community | 6, 749 | 8.923 | **>0.0001** |
|  | Nitrogen x Light | 1, 749 | 0.454 | 0.5007 |
|  | Nitrogen x Soil | 2, 749 | 0.928 | 0.3959 |
|  | Nitrogen x Community | 6, 749 | 0.441 | 0.8516 |
|  | Light x Soil | 2, 749 | 0.099 | 0.9055 |
|  | Light x Community | 6, 749 | 1.867 | 0.0840 |
|  | Soil x Community | 12, 749 | 2.738 | **0.0012** |
| Peak Biomass |  |  |  |  |
|  | Nitrogen | 1, 749 | 0.602 | 0.4381 |
|  | Light | 1, 749 | 7.475 | **0.0064** |
|  | Soil | 2, 749 | 5.484 | **0.0043** |
|  | Community | 6, 749 | 2.769 | **0.0114** |
|  | Nitrogen x Light | 1, 749 | 2.651 | 0.1039 |
|  | Nitrogen x Soil | 2, 749 | 0.649 | 0.5227 |
|  | Nitrogen x Community | 6, 749 | 1.836 | 0.0894 |
|  | Light x Soil | 2, 749 | 1.269 | 0.2818 |
|  | Light x Community | 6, 749 | 1.256 | 0.2753 |
|  | Soil x Community | 12, 749 | 1.816 | **0.0420** |

Significant factors (P<0.05) are in bold. All possible interactions were included in statistical model, but non-significant 3 and 4-way interactions are not shown.
