# Supplementary material for: The Effect of Nitrogen Deposition on Plant Performance and Community Structure: Is It Life Stage Specific?
Source: PLoS One. 2016 Jun 2;11(6):e0156685. doi: 10.1371/journal.pone.0156685 (PMC4890792; doi:10.1371/journal.pone.0156685)
Supplement: S5 Table — Significant factors (P<0.05) are in bold. All possible interactions were included in statistical model, but non-significant 3 and 4-way interactions are not shown. (DOCX) [file pone.0156685.s010.docx]

**S5 Table. 4-way ANOVA and MANOVA (N x Soil x Light x Community) F statistics for naturalized exotic seed mass and number during the senescent stage.**

|  | ANOVA | | | MANOVA | |
| --- | --- | --- | --- | --- | --- |
| Factor | df | Seed Number (g/ind.) | Seed Mass (g/ind.) | df | Wilks Lambda |
| Nitrogen | 1 | 0.4028 | 0.2937 | 4 | 0.4208 |
| Light | 1 | 0.0062 | 5.4880* | 4 | 2.7907 |
| Soil | 2 | 11.0932*** | 0.3059 | 8 | 5.4528*** |
| Community | 3 | 6.8598*** | 2.0747 | 12 | 3.8843*** |
| Nitrogen x Light | 1 | 0.3013 | 8.1378** | 4 | 4.0526* |
| Nitrogen x Soil | 2 | 0.4579 | 0.2313 | 8 | 0.3106 |
| Nitrogen x Community | 6 | 0.8424 | 1.0425 | 12 | 0.8599 |
| Light x Soil | 2 | 1.0538 | 0.2777 | 8 | 0.6231 |
| Light x Community | 3 | 0.6700 | 0.7579 | 12 | 0.7150 |
| Soil x Community | 6 | 1.8606 | 1.8226 | 24 | 1.6079 |

MANOVA used Wilk’s Lambda to calculate F statistic and p-values. All possible interactions were included in statistical model, but non-significant 3 and 4-way interactions are not shown.

*P<0.05

**P<0.01

***P<0.001
